# Alveolar macrophages regulate neutrophil recruitment in endotoxin-induced lung injury

**DOI:** 10.1186/1465-9921-6-61

**Published:** 2005-06-22

**Authors:** Beatrice Beck-Schimmer, Reto Schwendener, Thomas Pasch, Livia Reyes, Christa Booy, Ralph C Schimmer

**Affiliations:** 1 Institute of Anesthesiology, University of Zurich, Switzerland; 2 Institute of Physiology, University of Zurich, Switzerland; 3 Paul Scherrer Institute, Villigen, Switzerland; 4 Department of Surgery, University of Zurich, Switzerland

**Keywords:** effector cells, lipopolysaccharide, lung inflammation, macrophage depletion, monocyte chemoattractant protein-1

## Abstract

**Background:**

Alveolar macrophages play an important role during the development of acute inflammatory lung injury. In the present study, *in vivo *alveolar macrophage depletion was performed by intratracheal application of dichloromethylene diphosphonate-liposomes in order to study the role of these effector cells in the early endotoxin-induced lung injury.

**Methods:**

Lipopolysaccharide was applied intratracheally and the inflammatory reaction was assessed 4 hours later. Neutrophil accumulation and expression of inflammatory mediators were determined. To further analyze *in vivo *observations, *in vitro *experiments with alveolar epithelial cells and alveolar macrophages were performed.

**Results:**

A 320% increase of polymorphonuclear leukocytes in bronchoalveolar lavage fluid was observed in macrophage-depleted compared to macrophage-competent lipopolysaccharide-animals. This neutrophil recruitment was also confirmed in the interstitial space. Monocyte chemoattractant protein-1 concentration in bronchoalveolar lavage fluid was significantly increased in the absence of alveolar macrophages. This phenomenon was underlined by *in vitro *experiments with alveolar epithelial cells and alveolar macrophages. Neutralizing monocyte chemoattractant protein-1 in the airways diminished neutrophil accumulation.

**Conclusion:**

These data suggest that alveolar macorphages play an important role in early endotoxin-induced lung injury. They prevent neutrophil influx by controlling monocyte chemoattractant protein-1 production through alveolar epithelial cells. Alveolar macrophages might therefore possess robust anti-inflammatory effects.

## Introduction

Lipopolysaccharide (LPS) is a component of the outer membrane of gram-negative bacteria, capable of inducing severe lung injury in sepsis or bacterial pneumonia upon release. Both conditions are predisposing causes of the acute respiratory distress syndrome (ARDS). Instillation of LPS into the lungs results in an acute inflammatory response orchestrated by the coordinated function of cytokines, chemokines, and adhesion molecules. *In vitro *and *in vivo *models of acute lung inflammation have been extensively used to elucidate the molecular mechanisms in endotoxin-induced lung injury [1-3]. However, only sparse data exist about the exact role of effector cells such as alveolar macrophages (AM) in the respiratory compartment in this type of injury.

The airway compartment with alveolar macrophages and epithelial cells as the predominant cell types is a physiological barrier to a variety of environmental agents including gases, particulates and microbes. An injury to lung tissue involving the airway compartment can lead to serious illness and life-threatening conditions such as ARDS. AM are located at the air-tissue interface in the lung and are therefore the first cells, which interact with inhaled organisms and antigens [4]. During the development of an acute inflammatory lung injury, AM play a very important role upon their activation. It has been shown in several lung injury models that activated pulmonary macrophages release the cytokines tumor necrosis factor-α (TNF-α) and interleukin-1β (IL-1β) as well as the chemokines monocyte chemoattractant protein-1 (MCP-1) and macrophage inflammatory protein-1β (MIP-1β) [5]. Intercellular adhesion molecule-1 (ICAM-1, CD54) and vascular cell adhesion molecule-1 (VCAM-1, CD106) are adhesion molecules of the immunoglobulin superfamily. Adhesion to vascular endothelium mediated through ICAM-1/VCAM-1-integrin interactions is a key step in emigration of white blood cells to sites of inflammation [6]. It has also been shown that they play a central role in adherence of effectors cells such as AM to the respiratory epithelium [7]. All these inflammatory mediators together play a crucial role in the orchestration of an inflammatory response, particularly in neutrophil recruitment. Recent studies suggest that cytokines and chemokines do not only originate from AM, but also from other cells such as epithelial cells [8].

In order to study the role and function of AM regarding inflammatory mediators in endotoxin-induced lung injury, AM were depleted by intratracheal application of dichloromethylene diphosphonate-(Cl_2_MDP)-liposomes (clodronate liposomes) as phagolysozymes. Phagocytosis of clodronate-liposomes has been shown to result in the selective destruction of macrophages with an elimination rate up to 80% [9]. Cellular and molecular immunopathologic characteristics in endotoxin-induced lung injury of non-depleted and AM-depleted animals were investigated, focusing on neutrophil recruitment and expression of inflammatory mediators. The hypothesis was tested if AM upon LPS stimulation have an anti-or proinflammatory character.

## Materials and methods

### Animals

Male pathogen-free 300 g Wistar rats (RCC Ltd, Füllinsdorf, Switzerland) were anesthetized with subcutaneous fentanyl-fluanisone (0.25 ml/kg) and medetomidine hydrochloridum (0.25 ml/kg). The animal care committees at the University of Zurich approved the experimental protocols.

### Clodronate Liposomes

Clodronate liposomes were prepared as described [10]. To verify any possible contamination with LPS, endotoxin levels were measured using an *in vitro *analysis of endotoxin (Limulus-Test; Endotell, Allschwil, Switzerland). With the negative results of these tests any biologically relevant contamination with LPS could be ruled out. Clodronate is a synthetic bisphosphonate which has been used to selectively eliminate macrophages in various tissues. Uptake of liposomal-encapsulated clodronate has been demonstrated by macrophages and monocytes, undergoing apoptosis in response to clodronate. Each animal received a dose of 500 μg liposome-encapsulated clodronate. Empty liposomes were used as controls. Liposomes were diluted in 300 μl phosphate-buffered saline (PBS) and instilled into the trachea of the animals.

### LPS Lung Injury

72 hours after liposome application animals were anesthetized again and lung injury was induced by intratracheal application of 150 μg LPS (Escherichia coli serotype 055:B5; Sigma Chemical Co, St. Louis, MO) in 300 μl PBS. Animals were sacrificed 4 hours or 24 hours later and lungs were analyzed.

For inhibition experiments rats received 100 μg intratracheally applied polyclonal neutralizing rabbit anti-rat MCP-1 antibodies or control IgG (both from Torrey Pines Biolabs, Houston, TX) 30 minutes after intratracheal instillation of LPS or PBS.

### Bronchoalveolar Lavage Fluid (BALF)

Bronchoalveolar lavage was performed with 10 ml cold PBS. BALF was centrifuged and the supernatant aliquoted and stored at -20°C. Cells were counted and differentiated using a cytospin and Diff-Quick staining (Dade Behring AG, Düdingen).

### Myeloperoxidase (MPO) Assay

In order to determine the parenchymal content of neutrophils, a MPO assay was performed as previously described [11]. The vascular and respiratory compartment were flushed with PBS in order to eliminate blood cells, notably neutrophils.

### ELISA Quantitation of TNF-α, MCP-1, and Chemokine-Inducible Neutrophil Chemoattractant-1 (CINC-1) in BALF

TNF-α, MCP-1, and CINC-1 were assessed in the BALF of animals using a standard enzyme-linked immunosorbent assay (ELISA) purchased from PharMingen, San Diego, CA (TNF-α and MCP-1) and from R&D Systems, Minneapolis, MN (CINC-1 Duoset).

### Western Blot Analysis of TNF-α, MCP-1, MIP-1β, and Macrophage Inflammatory Protein-2 (MIP-2) in BALF

BALF of animals were loaded and electrophoresed in a 7.5% SDS-polyacrylamide gel. After separation, the proteins were transblotted to a nitrocellulose membrane. Incubation with a polyclonal goat anti-rat TNF-α, anti-rat MCP-1, and anti-MIP-2 antibody (all from Santa Cruz Biotechnology, Santa Cruz, CA) or with a polyclonal rabbit anti-rat MIP-1β (Santa Cruz Biotechnology, Santa Cruz, CA) was performed overnight at 4°C. A secondary horseradish peroxidase-labeled anti-goat or anti-rabbit IgG (1:5000) was added for 30 minutes at room temperature. Signals were detected by enhanced chemiluminescence.

### Isolation and Reverse Transcription PCR Amplification of Lung mRNA

The vascular and respiratory compartments were flushed before using the lungs for determination of mRNA of various genes. Total RNA from lungs was extracted as previously described and analyzed by Reverse Transcription Polymerase Chain Reaction (RT-PCR) (Perkin-Elmer, Branchburg, NJ) [7]. The primers used for gene analysis are summarized in Tab. 1. PCR was also performed with 18S primers to ensure equal loading and mRNA/18S ratios were calculated.

**Table 1:** Optimized conditions for RT-PCR

			Size of PCR	
Gene	Primers		Fragments (bp)	Thermocycle Condition
TNF-α	sense	5'-ACT GAA CTT CGG GGT GAT TG-3'	334	23 cycles; T_m_: 57°C
	antisense	5'-GTG GGT GAG GAG CAG GTA GT-3'		
				
IL-1β	sense	5'-AGC TGC ACT GCA GGC TTC GAG ATG-3'	339	28 cycles; T_m_: 68°C
	antisense	5'-GAA CTG TGC AGA CTC AAA CTC CAC-3'		
				
MCP-1	sense	5'-TAT GCA GGT CTC TGT CAC GC-3'	255	24 cycles; T_m_: 58°C
	antisense	5'-TTC CTT ATT GGG GTC AGC AC-3'		
				
MIP-1β	sense	5'-CGT GTC TGC CTT CTC TCT CC-3'	220	24 cycles; T_m_: 58°C
	antisense	5'-CAC AGA TTT GCC TGC CTT TT-3'		
				
ICAM-1	sense	5'-AGG TAT CCA TCC ATC CCA CA-3'	660	22 cycles; T_m_: 58°C
	antisense	5'-CTT CAG AGG CAG GAA ACA GG-3'		
				
VCAM-1	sense	5'-CGG TCA TGG TCA AGT GTT TG-3'	570	24 cycles; T_m_: 57°C
	antisense	5'-ACC CTC ATG TAG CCT TGT GG-3'		

### *In vitro *Assay with Alveolar Epithelial Cells (AEC)

AEC from rat lungs were collected following previously described protocols [12]. Cell purity of AEC was more than 95%. AM were harvested following an earlier protocol [7]. AEC were kept for 5 days in culture. Most of the cells showed type I phenotype. At the time of co-culture with AM, AEC were confluent. To each well of a 96-well plate with AEC 1 × 10^7 ^AM were added. The relation of AM to AEC was 1:2 (1 × 10^7 ^and 2 × 10^7^), when the experiments were performed, according to previous studies [13]. AM were allowed to settle on AEC for 30 min prior to the addition of LPS. As a control AEC and AM were stimulated separately. Supernatants were collected and ELISA (TNF-α, MCP-1) or Western blot (MIP-1β) was performed as described above.

### Chemotaxis Assays

1 × 10^5 ^polymorphonuclear cells (PMN) of calcein-AM labelled neutrophils were given into MultiScreen-MIC filter plates [14]. Receiver plates were loaded with 150 μl of BALF. To some of these wells, 10 μg/ml polyclonal neutralizing rabbit anti-rat MCP-1 (Torrey Pines Biolabs, Houston, TX) or 10 μg/ml control IgG (Torrey Pines Biolabs, Houston, TX) were added. For experiments with CINC-1 antibodies 10 μg/ml monoclonal mouse anti-rat CINC-1 antibody (R&D Systems, Europe Ltd., Abingdon, Oxon, UK) or 10 μg/ml MOPC-21 as control (mouse IgG, Pharmingen, San Diego, CA) were used. 0.1 μM of the chemotactic substance fMLP (Sigma-Aldrich, Buchs, Switzerland) was taken as positive control and PBS/1% fetal bovine serum (FBS) to determine basal migration. After 2 hours incubation at 37°C the filter was removed and migrated cells were lysed with Triton 1% (Sigma-Aldrich, Buchs, Switzerland). Fluorescence was measured by using an excitation filter at 485 nm and an emission filter at 535 nm. Ratio between stimulated and basal migration was used as indication of PMN chemotaxis.

### Statistical Methods

For each group and/or time point 5 animals were used. The relative reduction in mRNA or protein expression was calculated for LPS clodronate-liposome results. LPS control liposome and LPS clodronate-liposome values were calculated relative to their respective PBS liposome values. LPS clodronate-liposome reduction or upregulation was calculated as the relative change compared to LPS control liposome results. ELISA contained three replicates with five different animals. Each data point in the graphs represents mean +/- standard error of mean (SEM). Analysis of variance (ANOVA) with post-ANOVA comparison was performed to assess the statistical significance of differences.

## Results

### Macrophage Depletion

Before experiments with clodronate-liposomes were started, different depletion conditions were evaluated as described [15]. Optimal depletion was seen at a dose of 500 μg for 72 hours. Depletion was verified with macrophage staining in BALF showing a depletion of 75–80%, which was consistent with previous reports [16]. Normal rats, not treated with liposomes, had 1.6 × 10^6 ^cells (all macrophages) in BALF. 72 hours after liposome instillation, cell count in BALF of control liposome animals showed 2.2 × 10^6 ^cells (all macrophages), while cells of BALF of clodronate-liposome pretreated animals had 1.0 × 10^6 ^cells (0.5 × 10^6 ^macrophages and 0.5 × 10^6 ^neutrophils). The phenomenon of a slight neutrophil recruitment induced by clodronate is a known observation [17].

### Detection of Cell Types in BALF and Determination of Interstitial PMN Recruitment

**Figure 1:**
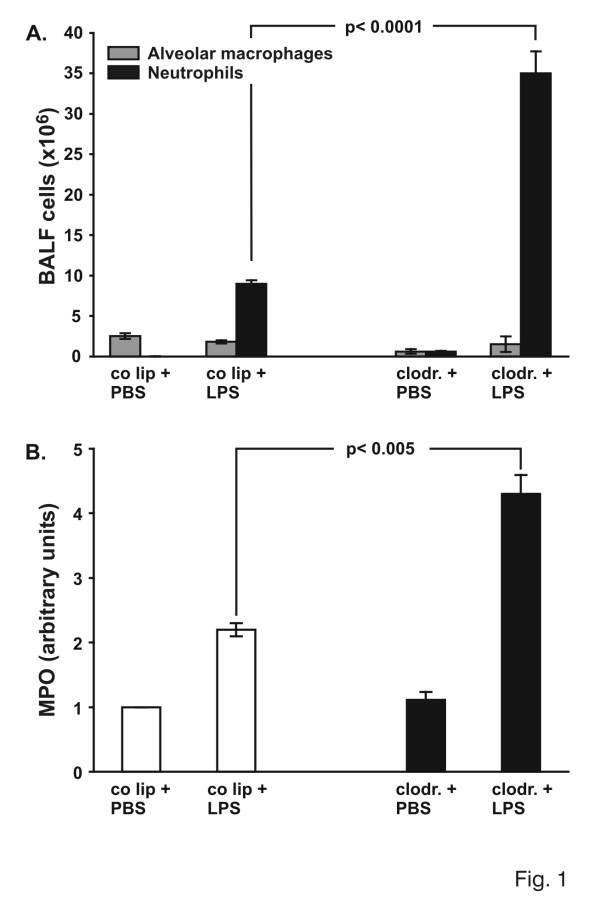
A: Total cell count in bronchoalveolar lavage fluid (BALF). Animals were pretreated with control liposomes (co lip) or clodronate-liposomes (clodronate). 72 hours later 150 μg LPS was instilled intratracheally and lungs were evaluated 4 hours later. Cells were analyzed using cytospin and Diff-Quick staining. Values are mean ± SEM from 5 animals per group. B. Determination of myeloperoxidase (MPO) activity as a measure of parenchymal neutrophil content. Animals were pretreated with control liposomes (co lip) or clodronate-liposomes (clodronate). 72 hours later 150 μg LPS was instilled intratracheally and lungs were evaluated 4 hours later. The right lung of each animal was removed and homogenized in sample buffer. Optical density was measured at 420 nm over 360s. The value for control liposomes and PBS was defined as 1 and all other values were adapted. Values are mean ± SEM from 5 animals per group.

Cells from control-and clodronate-liposome animals, receiving PBS or LPS, were analyzed in BALF (Fig. 1A). Total cell count in BALF of control liposome animals with intratracheal PBS was 2.5 × 10^6 ^cells (all macrophages). In PBS-animals pretreated with clodronate-liposomes, cell count decreased to 1.2 × 10^6 ^cells (0.6 × 10^6 ^macrophages, 0.6 × 10^6 ^neutrophils). BALF of control liposome-animals, receiving LPS, showed 10.0 × 10^6 ^cells (4-fold increase), while in clodronate-animals with LPS, 36.5 × 10^6 ^cells were detected in BALF. The difference between LPS-animals with and without AM was 320% (p < 0.0001). The absolute increase of AM from 0.6 × 10^6 ^cells in clodronate-PBS animals compared to clodronate-LPS animals with 1.5 × 10^6 ^cells might be due to a recruitment of interstitial macrophages.

To determine whether the amount of interstitial neutrophils changed between AM-competent and AM-depleted animals, lungs were harvested 4 hours after intratracheal application of PBS or LPS. The vascular and airway compartment were flushed, and MPO activity was determined (Fig. 1B). Results of AM-competent PBS-animals were defined as 1, while other results were adapted. Intratracheal application of LPS resulted in a 115% enhancement of MPO. A 220% increase in lung MPO activity was observed in LPS-animals without AM as compared to AM-competent animals (p < 0.005).

### TNF-α, MCP-1, MIP-1β, MIP-2, and CINC-1 Protein Determination in BALF

The assessment of inflammatory cells showed a significant increase of neutrophils in AM-depleted LPS-animals as compared to non-depleted LPS-animals. Therefore, three potentially activating and chemotactic cytokines and chemokines in the BALF were examined: TNF-α, MCP-1 and MIP-1β.

As shown by ELISA in Tab. 2 TNF-α was not detectable in control-and clodronate-liposome animals treated with PBS. However, TNF-α concentration in control liposome LPS-animals increased up to 3.4 ng/ml, while AM-depleted LPS-animals had only 1 ng/ml TNF-α (71% decrease, p < 0.001). Therefore, AM – rather than the increased number of neutrophils – appear to be a major source of TNF-α protein in BALF of LPS-injured lungs. Contrary to TNF-α, MCP-1 protein in LPS-injured animals increased from 8.0 ng/ml to 21.5 ng/ml upon AM depletion (114% increase, p < 0.0001) (Tab. 2), indicating that the presence of AM suppresses MCP-1 production or that enhanced neutrophil influx may be responsible for this phenomenon of increased MCP-1 protein concentration.

**Table 2:** Determination of TNF-α and MCP-1 in BALF by ELISA

**TNF-α (ng/ml)**		
		
	**4 h**	**24 h**
		
co lip + PBS	0.07 +/- 0.01	0.03 +/- 0.06
co lip + LPS	3.37 +/- 0.07	1.01 +/- 0.17
		
clodr. + PBS	0.04 +/ -0.00	0.07 +/- 0.11
clodr. + LPS	1.08 +/- 0.39*	1.12 +/- 0.13 n.s.
**MCP-1 (ng/ml)**		
		
	**4 h**	**24 h**
		
co lip + PBS	0.44 +/- 0.09	0.57 +/- 0.45
co lip + LPS	8.16 +/- 0.63	13.50 +/- 2.94
		
clodr. + PBS	5.00 +/- 0.31	2.07 +/- 1.05
clodr. + LPS	21.5 +/- 1.80**	11.13 +/- 2.76 n.s.

* p < 0.001, **p < 0.0001 clodr. LPS compared to co lip LPS

**Figure 2:**
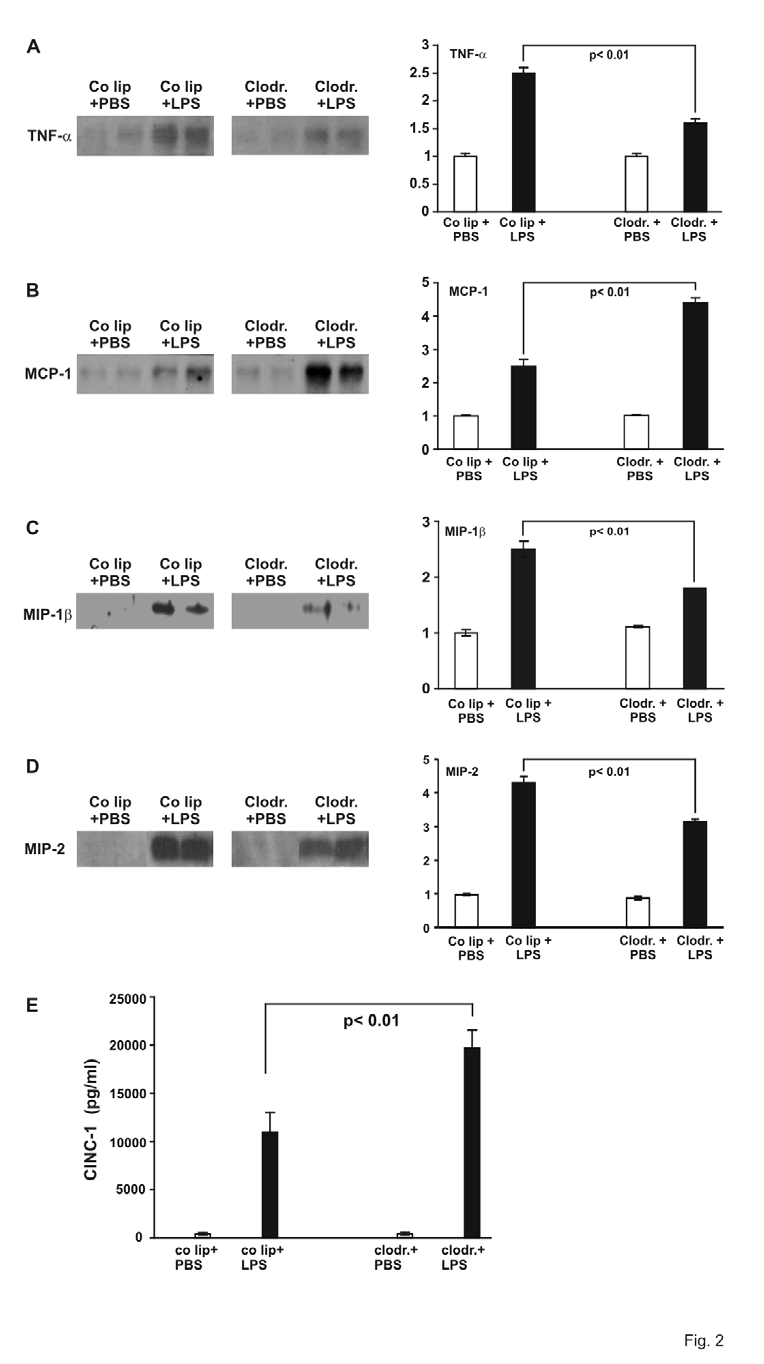
A. TNF-α and protein determination in bronchoalveolar lavage fluid of AM-competent (control liposome = co lip) and AM-depleted (clodronate) animals, exposed to PBS or LPS for 4 hours. Proteins were electorphoresed on a SDS-polyacrylamide gel and transblotted to a nitrocellulose membrane. Densitometry was performed. One value for control liposomes and PBS was defined as 1 and all other values were adapted. Values are mean ± SEM from 5 animals (only 2 are shown). B. MCP-1 protein determination in bronchoalveolar lavage fluid of AM-competent and -depleted animals, exposed to PBS or LPS for 4 hours. Proteins were electorphoresed on a SDS-polyacrylamide gel and transblotted to a nitrocellulose membrane. Densitometry was performed. One value for control liposomes and PBS was defined as 1 and all other values were adapted. Values are mean ± SEM from 5 animals (only 2 are shown). C. MIP-1β protein determination in bronchoalveolar lavage fluid of AM-competent and -depleted animals, exposed to PBS or LPS for 4 hours. Proteins were electorphoresed on a SDS-polyacrylamide gel and transblotted to a nitrocellulose membrane. Densitometry was performed. One value for control liposomes and PBS was defined as 1 and all other values were adapted. Values are mean ± SEM from 5 animals (only 2 are shown). D. MIP-2 protein determination in bronchoalveolar lavage fluid of AM-competent and -depleted animals, exposed to PBS or LPS for 4 hours. Proteins were electorphoresed on a SDS-polyacrylamide gel and transblotted to a nitrocellulose membrane. Densitometry was performed. One value for control liposomes and PBS was defined as 1 and all other values were adapted. Values are mean ± SEM from 5 animals (only 2 are shown). E. CINC concentration in bronchoalveolar lavage fluid (BALF) of AM-competent (control liposomes = co lip) and AM-depleted (clodronate) animals, exposed to PBS or LPS for 4 hours. CINC was examined using a standard ELISA. Values are mean ± SEM from 5 animals.

As liposomes might interact with an ELISA technique and thus lead to an incorrect result, BALF was also analyzed by Western blot technique for TNF-α, MCP-1, and MIP-1β concentrations (Fig. 2). TNF-α protein levels in BALF decreased upon LPS stimulation in AM-depleted animals compared to LPS-stimulated AM-competent animals (60% decrease, p < 0.01, Fig. 2A). MCP-1 protein concentration was enhanced in AM-depleted LPS animals (126% increase, p < 0.01, Fig. 2B). Therefore, the results were comparable with ELISA data. For MIP-1β, a decrease of 53% was seen in AM-depleted LPS animals compared to AM competent LPS animals (p < 0.01, Fig. 2C).

As it was unlikely that MCP-1 was the only PMN chemoattractant further investigations were performed focusing on MIP-2 and CINC-1. MIP-2 showed an increase of 230% in control liposome LPS animals compared to PBS animals. 32% less protein were detected in clodronate LPS animals than in control liposome LPS ones (p < 0.01) (Fig. 2D). CINC-1 protein, however, showed a similar increase as MCP-1 upon LPS stimulation in AM-depleted animals compared to LPS animals with AM: it increased from 10.9 ng/ml in control liposome LPS-animals to 19.7 ng/ml in clodronate LPS-animals, 86% increased, p < 0.01; Fig. 2E). Therefore, it could be considered as a chemoattractant for PMN.

### Cytokine, Chemokine, and Adhesion Molecule mRNA Expression in Rat Lung

**Figure 3:**
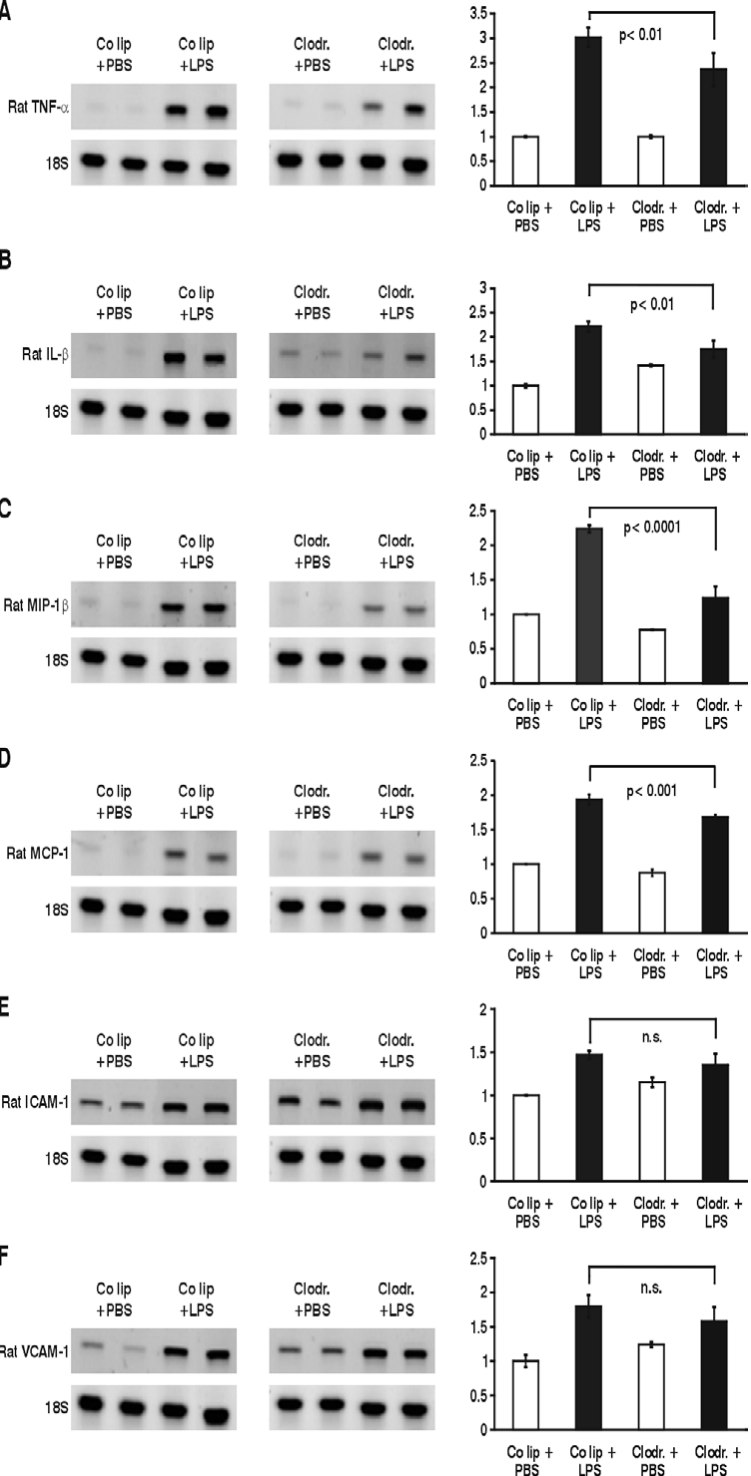
Changes in expression of TNF-α mRNA (A), IL-1β mRNA (B), MIP-1β mRNA (C), MCP-1 mRNA (D), ICAM-1 mRNA (E) and VCAM-1 mRNA (F) of AM-competent (control liposomes = co lip) and -depleted (clodrondate) animals after 4 hours of PBS/LPS stimulation. Whole lung RNA was extracted and RT-PCR performed according to the primers and annealing thermocycle conditions shown in Tab. 1. Equal loading was shown with 18S bands. Results were analyzed by densitometry. One value for control liposomes and PBS was defined as 1 and all other values were adapted. Values are mean ± SEM from 5 animals.

To obtain more information on cytokine and chemokine levels in the respiratory compartment of whole lungs, mRNA was isolated from lungs 4 hours after airway instillation of PBS or LPS in control liposome-and clodronate-liposome animals (previously lavaged lungs). The presence of cytokines, chemokines and adhesion molecules was assessed by reverse transcription-PCR (Fig. 3A–F). No constitutive mRNA expression for TNF-α, IL-1β, MIP-1β, MCP-1 was detectable in lung tissue of control liposome animals, which received PBS. After instillation of LPS in control liposome animals enhanced mRNA expression of the analyzed cytokines and chemokines was demonstrated. In LPS-stimulated AM-depleted animals, TNF-α decreased by 32% (p < 0.01) in comparison to LPS-stimulated control liposome animals, while IL-1β was decreased by 73% (p < 0.01). For the chemokines MIP-1β and MCP-1, a reduction of 62%, respectively 14%, was observed in mRNA expression in LPS-injured AM-depleted animals compared to control liposome animals stimulated with LPS (p < 0.0001, resp. p < 0.001). Only a minor expression of ICAM-1-mRNA and VCAM-mRNA was seen in control liposome PBS-animals. mRNA increased in LPS-injured AM-competent and AM-depleted animals compared to those animals receiving PBS. However the difference between AM-competent and AM-depleted LPS-animals did not reach statistical significance.

### TNF-α-, MCP-1-, and MIP-1β Production in AEC and AM in Co-Culture

**Figure 4:**
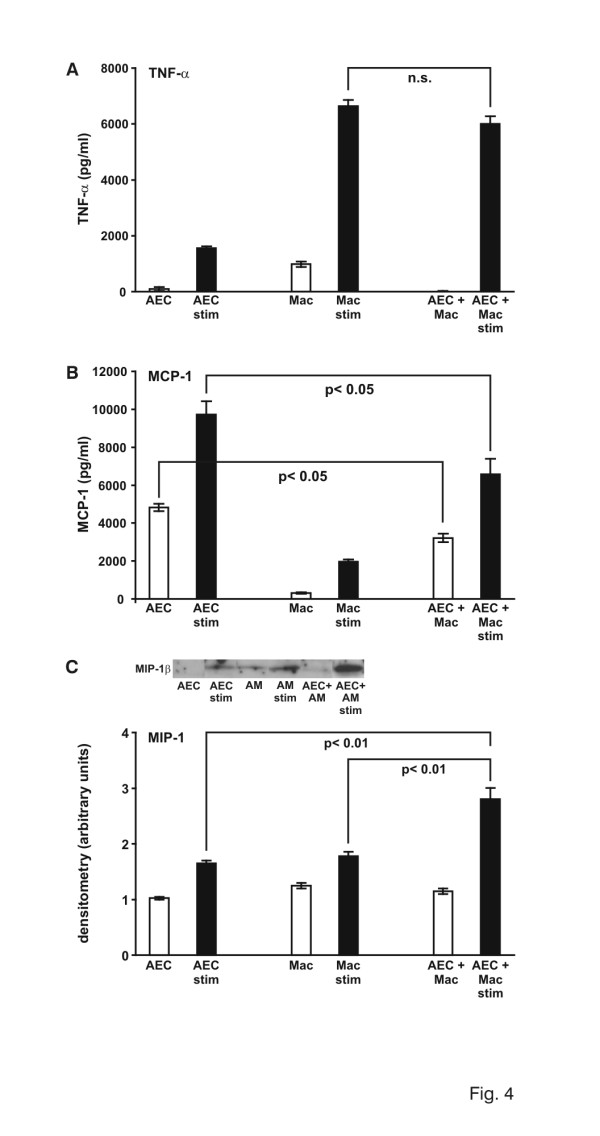
A. TNF-α protein determination in supernatants of alveolar epithelial cells (AEC), alveolar macrophages (AM) and co-culture of AEC/AM upon LPS stimulation (relation AEC:AM = 2:1). TNF-α was examined using a standard ELISA. White bars represent control cells, black bars LPS-stimulated cells. Values are mean ± SEM from 5 different assays. B. MCP-1 protein determination in supernatants of alveolar epithelial cells (AEC), alveolar macrophages (AM) and co-culture of AEC/AM upon LPS stimulation (relation AEC:AM = 2:1). MCP-1 was examined using a standard ELISA. White bars represent control cells, black bars LPS-stimulated cells. Values are mean ± SEM from 5 different assays. C. MIP-1β protein determination in supernatants of alveolar epithelial cells (AEC), alveolar macrophages (AM) and co-culture of AEC/AM upon LPS stimulation (relation AEC:AM = 2:1). MIP-1β was examined using Western blot analysis. Densitometry was performed. One value for control liposomes and PBS was defined as 1 and all other values were adapted. White bars represent control cells, black bars LPS-stimulated cells. Values are mean ± SEM from 5 different assays.

*In vitro *assays were performed to further explore the interactions of AEC and AM in the respiratory compartment, observed *in vivo*. In a first set of experiments, optimal conditions were evaluated testing different seeding densities and LPS concentrations (data not shown). AEC were co-incubated with AM at a relation of 2:1 (AEC:AM) and stimulated with 10 μg/ml LPS. Upon stimulation, TNF-α production in AM increased from 0.98 ng/ml to 6.6 ng/ml (p < 0.001), while AEC under control conditions produced 0.08 ng/ml and 1.6 ng/ml after LPS stimulation (p < 0.001) (Fig. 4A). These *in vitro *results would support the *in vivo *observations of decreased TNF-α concentrations in BALF upon depletion of AM.

Unstimulated AEC showed a constitutive MCP-1 expression of 4.8 ng/ml, while MCP-1 concentration in the supernatant decreased to 3.2 ng/ml, when co-incubating AEC and AM (Fig. 4B). Upon LPS stimulation the following observations were made: AEC produced 9.7 ng/ml (p < 0.05), while in a system of AEC/AM co-incubation, MCP-1 protein concentration was only 6.6 ng/ml. This represents significantly less MCP-1 protein than produced by AEC in mono-culture (p < 0.05). These findings corroborate our *in vivo *data with less MCP-1 in the presence of AM.

Analyzing the supernatants for MIP-1β concentration, it was obvious that not only AM but also AEC produce MIP-1β upon LPS stimulation (Fig. 4C). However, in contrast to MCP-1 secretion, LPS-induced MIP-1β production was increased by 153% when co-incubating AEC and AM.

### Effect of Intratracheal Instillation of MCP-1 Antibody

**Figure 5:**
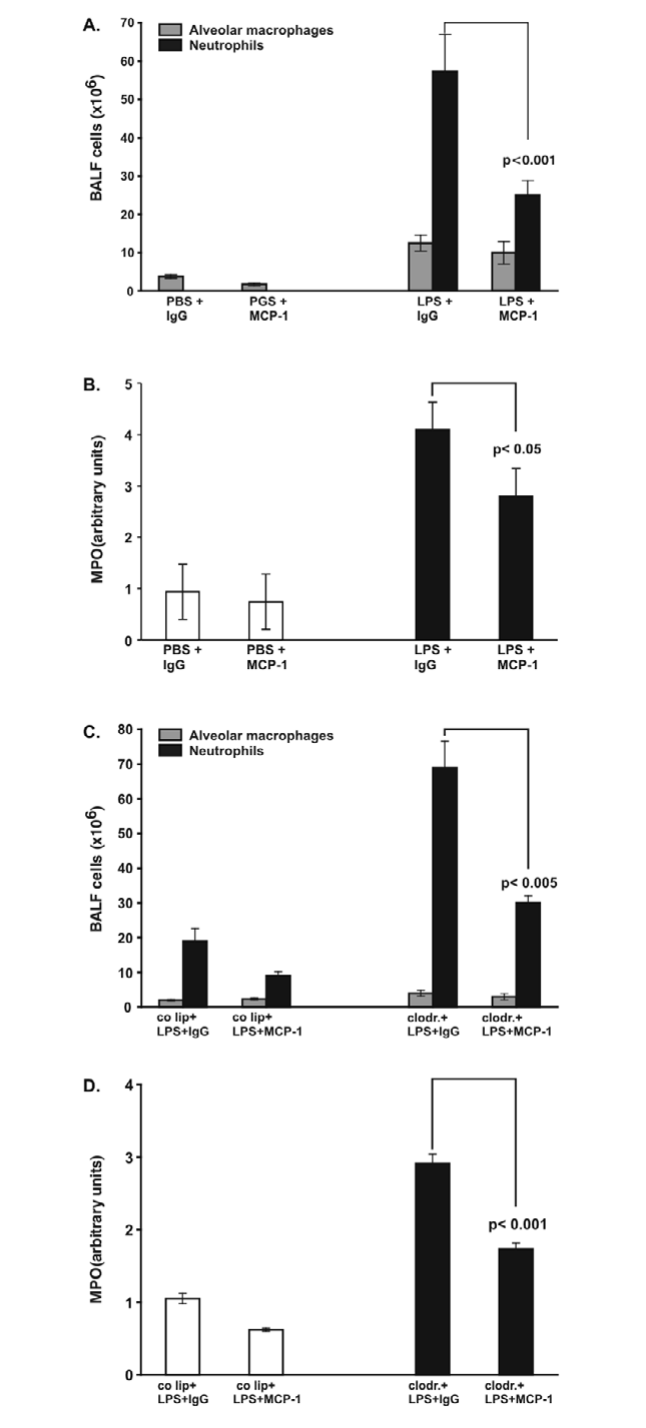
A. Total cell count in bronchoalveolar lavage fluid (BALF). Rats were challenged with intratracheal instillation of LPS or PBS, followed by intratracheal application of MCP-1 antibody or a control antibody (IgG). Lungs were evaluated 4 hours later. Cells were analyzed using cytospin and Diff-Quick staining. Values are mean ± SEM from 5 animals. B. Determination of myeloperoxidase (MPO) activity as a measure of parenchymal PMN content. Rats were challenged with intratracheal instillation of LPS or PBS, followed by intratracheal application of MCP-1 antibody or a control antibody (IgG). Lungs were evaluated 4 hours later. The right lung of each animal was removed and homogenized in sample buffer. Optical density was measured at 420 nm over 360s. One value PBS+IgG was defined as 1 and all other values were adapted. Values are mean ± SEM from 5 animals. C. Total cell count in bronchoalveolar lavage fluid (BALF). AM-competent (control liposomes = co lip) and -depleted (clodrondate) animals were challenged with intratracheal instillation of LPS, followed by intratracheal application of MCP-1 antibody or a control antibody (IgG). Lungs were evaluated 4 hours later. Cells were analyzed using cytospin and Diff-Quick staining. Values are mean ± SEM from 5 animals. D. Determination of myeloperoxidase (MPO) activity as a measure of parenchymal PMN content. AM-competent (control liposomes = co lip) and -depleted (clodrondate) animals were challenged with intratracheal instillation of LPS, followed by intratracheal application of MCP-1 antibody or a control antibody (IgG). Lungs were evaluated 4 hours later. The right lung of each animal was removed and homogenized in sample buffer. Optical density was measured at 420 nm over 360s. One value LPS+IgG was defined as 1 and all other values were adapted. Values are mean ± SEM from 5 animals.

Results of incrased PMN accumulation in the presence of MCP-1 raised the question if MCP-1 has neutrophil chemoattractant characteristics in this model. Therefore, an MCP-1-blocking antibody was directly instilled into the airways. Different amounts of antibody were tested (50 μg, 100 μg, and 150 μg). A maximal blocking effect on neutrophil accumulation was achieved with 100 μg. Increasing the amount of MCP-1 antibody beyond this dose did not result in a better blocking effect. Upon LPS stimulation PMN recruitment into the respiratory compartment was attenuated by 50% (p < 0.001) (Fig. 5A) in the presence of blocking antibodies, while interstitial PMN content was decreased by 35% (p < 0.05) (Fig. 5B).

The same experiments were performed in control lipsome-and clodronate-animals with LPS-induced lung injury. Similar results were found with 57% (p < 0.005) less neutrophils in the respiratory compartment (Fig. 5C) and 50% (p < 0.001) less interstitial PMN (Fig. 5D).

### Chemotactic Activity of BALF

**Figure 6:**
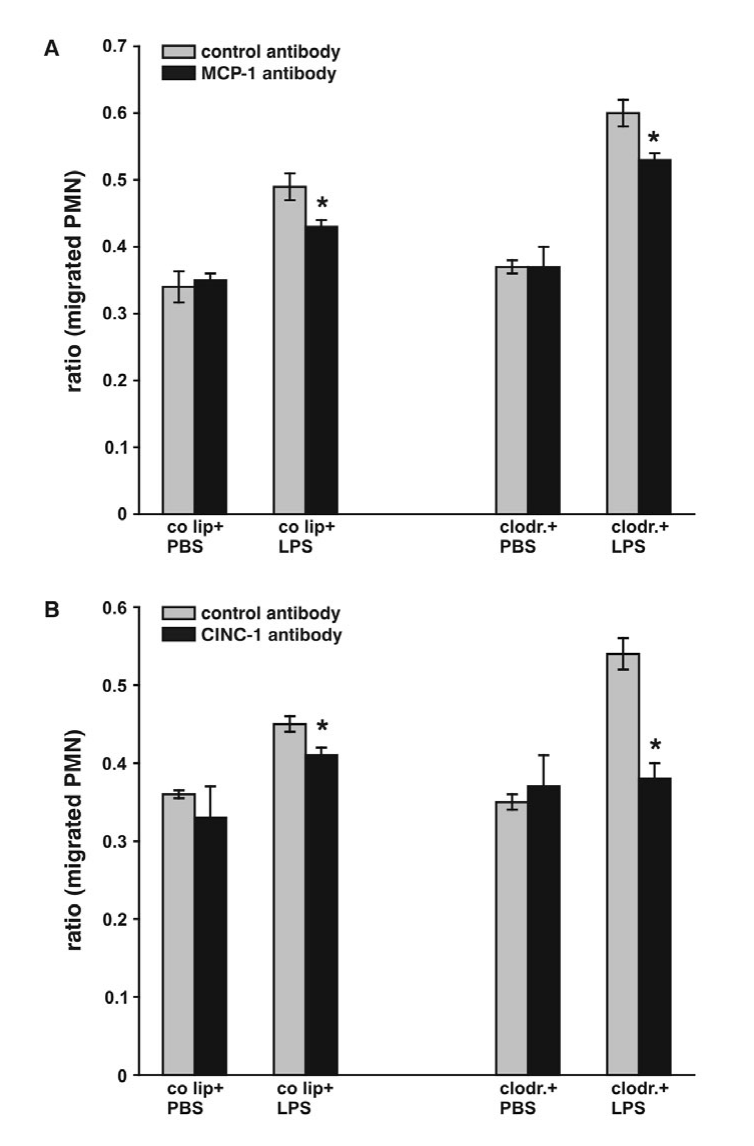
A. Chemotaxis assays performed with BALF of control liposome-PBS/LPS and clodronate-PBS/LPS animals. 1 × 10^5 ^polymorphonuclear cells (PMN) were labelled with calcein and given to the upper chamber, while the lower one was loaded with 150 μl of BALF. 10 μg/ml neutralizing anti-rat MCP-1 antibody or 10 μg/ml control IgG were added to BALF. PBS/1% fetal bovine serum was taken to determine basal migration. Migrated cells were lysed and fluorescence was measured by using an excitation filter at 485 nm and an emission filter at 535 nm. Ratio between stimulated and basal migration was used as indication of PMN chemotaxis. * p < 0.05 B. Chemotaxis assays performed with BALF of control liposome-PBS/LPS and clodronate-PBS/LPS animals. 1 × 10^5 ^polymorphonuclear cells (PMN) were labelled with calcein and given to the upper chamber, while the lower one was loaded with 150 μl of BALF. 10 μg/ml neutralizing anti-rat CINC-1 antibody or 10 μg/ml MOPC-21 were added to BALF. PBS/1% fetal bovine serum was taken to determine basal migration. Migrated cells were lysed and fluorescence was measured by using an excitation filter at 485 nm and an emission filter at 535 nm. Ratio between stimulated and basal migration was used as indication of PMN chemotaxis. * p < 0.05

To determine chemotactic activity in BALF of the different animal groups, chemotaxis assays were performed. In addition, the chemotactic activity of MCP-1 and CINC-1 was indirectly determined by using a neutralizing MCP-1 or CINC-1 antibody. Migration of PMN upon exposure to BALF from PBS animals with or without AM was similar (Fig. 6A and 6B). Upon LPS stimulation, it increased by 44% in the control liposome group (p < 0.05) and 76% in the clodronate group (p < 0.01). Notably, blocking MCP-1 in BALF with a neutralizing antibody decreased PMN migration (18% in control liposome-LPS animals, p < 0.05, 30% in clodronate-LPS animals, p < 0.05) (Fig. 6A). A 74% decrease of accumulation of neutrophils was found in the clodronate-LPS group using a blocking CINC-1 antibody (p < 0.05) (Fig. 6B).

### Effect of Alveolar Macrophage Depletion after 24 Hours of LPS Stimulation

**Figure 7:**
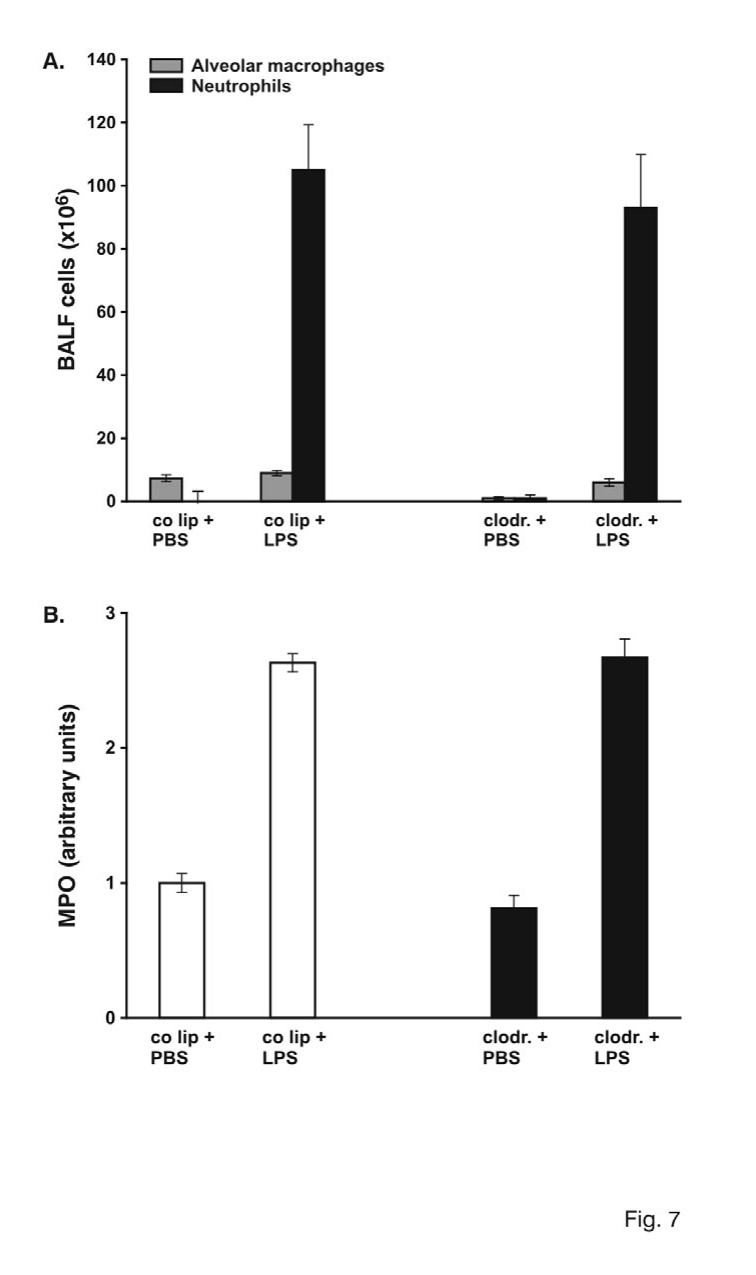
A: Total cell count in bronchoalveolar lavage fluid (BALF). Animals were pretreated with control liposomes (co lip) or clodronate-liposomes (clodronate). 72 hours later 150 μg LPS was instilled intratracheally and lungs were evaluated 24 hours later. Cells were analyzed using cytospin and Diff-Quick staining. Values are mean ± SEM from 5 animals. B. Determination of myeloperoxidase (MPO) activity as a measure of parenchymal neutrophil content. Animals were pretreated with control liposomes (co lip) or clodronate-liposomes (clodronate). 72 hours later 150 μg LPS was instilled intratracheally and lungs were evaluated 24 hours later. The right lung of each animal was removed and homogenized in sample buffer. Optical density was measured at 420 nm over 360s. One value for control liposomes and PBS was defined as 1 and all other values were adapted. Values are mean ± SEM from 5 animals.

To further verify the role of AM at a later time point of inflammation, LPS-injured lungs were analyzed after 24 hours. Interestingly, the impact of AM seemed to be different at 24 hours than at 4 hours. As shown in Fig. 7A cell recruitment was of similar magnitude in control liposome-and clodronate-animals: 114.3 × 10^6 ^cells in control liposome-LPS animals and 99.3 × 10^6 ^cells in BALF of clodronate-LPS animals (n.s.). A similar result with no difference between control lipsome-and clodronate-LPS group was seen in interstitial neutrophil recruitment (Fig. 7B). In addition to these findings, concentrations of TNF-α and MCP-1 in the respiratory compartment were not different in the two groups (Tab. 2). Furthermore there was no difference in whole lung mRNA of TNF-α, IL-1β, MIP-1β, MCP-1, ICAM-1, and VCAM-1 (data not shown).

## Discussion

AM are considered to play a predominant role in the inflammatory response to lung injury. An effective host defense is primarily dependent on this cell type [18]. AM do not only act as phagocytes, but have also a potent function as secretory cells. Upon stimulation, AM release a wide variety of biologically active molecules, which are part of the complex regulatory system of inflammatory reactions. A recent study demonstrated that depletion of AM in a murine model of *Klebsiella pneumoniae*-induced lung injury decreased survival dramatically by causing 100% lethality 3 days after infection, thereby demonstrating that AM exert a crucial role in this model [17]. In contrast to these findings, the elimination of AM promoted pulmonary immunity in a model of *Listeria*-induced lung injury [19]. To further examine the role of AM in the pathogenesis of neutrophilic lung inflammation in the early phase of LPS-induced lung injury, macrophages were eliminated by administration of liposome-encapsulated clodronate. Increased neutrophil recruitment was found in depleted LPS-injured animals compared to non-depleted LPS-injured animals. We postulated that beside other neutrophil chemoattractants enhanced concentrations of MCP-1 as detected in the respiratory compartment might be responsible for this increase in neutrophil recruitment. Therefore, AM could have an anti-inflammatory effect at this early time point of injury suppressing MCP-1 production by AEC.

Neutrophil accumulation in the lungs is one of the hallmarks of an acute inflammatory response. The mechanisms by which PMN are recruited into the lung in response to inflammatory stimuli are unclear, but seem to be dependent on the release of chemotactic and activating cytokines and chemokines [20]. These molecules recruit neutrophils and mediate their migration into the interstitial and finally into the alveolar space. Several models of lung injury have already been explored regarding neutrophil recruitment after AM-depletion. A model of AM-depletion with both intratracheally and intravenously delivered liposome-encapsulated clodronate showed decreased neutrophilic alveolitis in AM-depleted lungs after exposure to aerosolic LPS [21]. In acute lung injury induced by deposition of immunoglobulin G immune complexes, myeloperoxidase activity was decreased by 60% in AMdepleted lungs compared to AM-competent animals [16]. Similar results were seen in a model of *Pseudomonas aeruginosa *infection [22]. In a *Klebsiella *model of lung injury, an increased number of neutrophils in AM-depleted animals was observed after AM depletion [17]. This *K. pneumoniae*-induced PMN recruitment was shown to be TNF-α-mediated. In our model with an increase of PMN upon AM depletion, beside CINC-1, MCP-1 might be a possible neutrophil chemoattractant in the respiratory compartment. Chemotaxis assays underlined this hypothesis.

The role of MCP-1 regarding neutrophil accumulation in inflammatory processes is controversial. The nature of the injury is likely to determine the effect of MCP-1 on PMN. MCP-1 has recently been shown to play a certain role in PMN recruitment in various experimental systems [23-25]. Johnston et al. demonstrated an induction of neutrophil chemotaxis in mesenteric tissue upon MCP-1 superfusion. MCP-1 could also be shown to be involved in neutrophil accumulation during gastric ulceration. Vozzelli et al. performed blocking studies with anti-MCP-1 antibodies in newborn rats, exposed to hyperoxia [26]. Animals injected with 25 μg of anti-MCP-1 showed reductions in macrophage as well as neutrophil cell counts. Decreasing macrophage and monocyte activation by treatment with anti-MCP-1 might indirectly reduce neutrophil influx by decreasing a significant cellular source of neutrophil chemokines. However, this group showed as well an attenuation of PMN recruitment in hyperoxic rats, treated with 5 μg anti-MCP-1 antibody, with an unchanged number of alveolar macrophages, possibly demonstrating the effect of MCP-1 on neutrophil recruitment. Maus et al. postulated that JE/MCP-1 together with endotoxin, when co-appearing in the alveolar compartment elict an early phase of lung inflammatory injury with increased neutrophil recruitment [27]. Treatment of mice with only JE/MCP-1 induced a monocyte, but not a neutrophil influx into the bronchoalveolar compartment, with monocyte number peaking at 48 hours. Challenging mice with endotoxin in the presence of JE/MCP-1 led to an increase of neutrophils at 12 hours, while monocyte counts peaked at 48 hours. However, alveolar deposition of both MCP-1 and endotoxin was a prerequisite for the response as MCP-1 without accompanying injury did not provoke neutrophil emigration into the lung. Therefore, these studies do not fully underline a chemoattractant component of MCP-1 regarding PMN recruitment. Another study of Maus et al. reported decreased neutrophil and monocyte recruitment in alveolar macrophage-depleted animals upon combined JE/MCP-1/LPS treatment, also not supporting the concept of MCP-1 as a neutrophil chemoattractant [28]. Notabely, the earliest time point after LPS application the authors focused on was 6 hours, and never at 4 hours.

While our data demonstrated enhanced PMN accumulation in LPS-injured AM-depleted animals, Koay et al. showed decreased neutrophil recruitment upon LPS stimulation in intratracheally and intravenously applied clodronate mice at 4 hours of injury. However, there are certain essential differences regarding the animal model. 1) Mice instead of rats were used. 2) An intratracheal and intravenous depletion was performed in this study, while we used only the intratracheal one. 3) Intratracheally applied LPS was aerosolized, while we instilled LPS, dissolved in PBS. 4) Much higher doses of LPS were used in mice than in rats, which could also explain a more severe inflammation and therefore an immediate participation of extraalveolar macrophages in the lung. Noteworthy is the observation, that also in the study of Koay MCP-1 levels were increased in bronchoalveolar lavage fluid in LPS-clodronate animals compared to LPS-control liposome animals. Although the methodological approach by Koay et al. and our experimental systems appear similar, two clearly different models were used.

While in the presence of AM the amount of MCP-1 in BALF was low in LPS-injured lungs, it increased dramatically in the absence of AM, leading to the assumption that epithelial cells are responsible for the majority of MCP-1 content in BALF, although a receptor-induced ligand internalization can not be excluded. The production of MCP-1 by epithelial cells has been demonstrated previously [29]. Our *in vivo *data lead to the new hypothesis, that AEC in the presence of AM are modulated in their activity in producing MCP-1. We therefore performed *in vitro *assays with primary cultures of AEC and co-incubation of these cells with AM. After exposure to LPS, AEC/AM together produced less MPC-1 protein than LPS-stimulated AEC alone. This lead to the assumption that AM potentially exert a negative control effect on AEC. A similar observation was made with human bronchial epithelial cells (HBEC) and AM in culture, when exposed to ambient pollution particles [30]. While most of the inflammatory mediators such as TNF-α, IL-1β and IL-6 were significantly increased upon stimulation of the HBEC/AM-co-culture, MCP-1 production was decreased. This *in vitro *phenomenon could help to understand the increased levels of MCP-1 protein in the BALF of AM-depleted animals, although limitations of *in vitro *models are well known and results have to be interpreted with caution.

Contrary to MCP-1, TNF-α protein in BALF of AM-depleted LPS-animals was decreased significantly as well as whole lung mRNA of TNF-α. A study with hyperoxia in AM-depleted rats showed no significant differences in lung TNF-α content [31]. In contrast to the observation in our experiments in AM-depleted LPS-lungs, an increase of TNF-α in the AM-depleted *Klebsiella *pneumonia lungs was observed 2 days after the onset of the injury [17]. Several factors could explain these differing observations: 1. The difference of TNF-α content might reflect the difference in time points at which the lung injury was assessed. While TNF-α was evaluated at an early time point in the LPS-model, the analysis was performed in the *Klebsiella *model after 2 days. Kinetic expression of TNF-α is known to be time-variable [32]. 2. The mechanism of the inflammatory response to bacteria or toxins might bear different characteristics. 3. Alveolar macrophages have been shown to secrete large amounts of TNF-α, while interstitial macrophages are more efficient in releasing IL-1β and IL-6 [33]. This finding supports our observation with a markedly reduced content of TNF-α protein in BALF and moderate decrease of whole lung mRNA for TNF-α. In our *in vitro *data, AM are the dominant cells secreting TNF-α.

In the pulmonary tissue compartment – in contrast to the airway compartment – elimination of macrophages resulted in decreased production of mRNA for TNF-α, IL-1β, and MIP-1β in LPS-injured lungs, but only to a degree between 10 and 60%. These data suggest that other cells than AM contribute to tissue injury. The discrepant observation of minimal MCP-1 mRNA and protein decrease in whole lung and on the other side a MCP-1 increase in the BAL was observed in other models of lung injury [21]. The most likely explanation for this discrepancy is that the inflammatory reactions do not bear the same characteristics in different compartments of the lung. mRNA of the adhesion molecules ICAM-1 and VCAM-1 was not changed. Therefore, it can be concluded that AM do not affect the expression of these important inflammatory mediators. This is in accordance with previous data showing that whole lung ICAM-1 is regulated by about 80% through TNF-α [1]. In our studies, mRNA for TNF-α was only decreased by 32%, which might not be sufficient to elicit an ensuing change in ICAM-1 mRNA.

In order to define the role of AM at a later time point during LPS-induced lung injury, AM depletion was performed and lungs were collected 24 hours after LPS instillation. No difference in neutrophil recruitment between lungs with or without AM was observed. Two conclusions could be drawn from these experiments: 1) AM do not play the same critical functional role any more regarding PMN recruitment at this later time point of injury. As previously shown by Xing et al. AM play a key role in the very early LPS-induced lung inflammation [34]. 2) New AM were recruited during 24 hours of injury. Depletion of AM has been observed to persist for 5 days, followed by an increase of AM (unpublished data). These data were evaluated without application of any injury. In the present model, however, recruitment of new macrophages in response to the injury can be assumed.

The current study provides evidence for an important protective role of AM very early in the course of LPS-induced lung injury. AM were shown to affect neutrophil recruitment by reducing the accumulation of PMNs, most likely via suppression of alveolar epithelial MCP-1 production. This observation was surprising and not in accordance with our previously stated hypothesis. The precise role and the mechanisms of AM in the up-or downregulation of inflammatory mediators remain to be further investigated. Therapeutic options for the effective management of endotoxin-induced lung injury are still very limited. Therefore, the detailed exploration of an animal model of LPS-induced lung injury with regard to the role of macrophages will be helpful for the determination of the mechanistic and activation pathways.

## Authors' Contributions

BBS carried out animal studies and molecular biology studies and helped drafting the manuscript.

RS provided liposomes.

LR and CB performed molecular biology studies.

TP participated in the design of the study and helped drafting the manuscript.

RS and RCS was involved in the design and coordination of the study and helped drafting the manuscript.
